# Using AIE Luminogen for Long-term and Low-background Three-Photon Microscopic Functional Bioimaging

**DOI:** 10.1038/srep15189

**Published:** 2015-10-15

**Authors:** Zhenfeng Zhu, Chris W. T. Leung, Xinyuan Zhao, Yalun Wang, Jun Qian, Ben Zhong Tang, Sailing He

**Affiliations:** 1 State Key Laboratory of Modern Optical Instrumentation (Zhejiang University), Centre for Optical and Electromagnetic Research, Zhejiang Provincial Key Laboratory for Sensing Technologies, JORCEP (Sino-Swedish Joint Research Center of Photonics), Zhejiang University, Hangzhou, 310058, China; 2 Department of Chemistry, The Hong Kong University of Science & Technology, Clear Water Bay, Kowloon, Hong Kong, China; 3 Bioelectromagnetics Laboratory, Zhejiang University School of Medicine, Hangzhou 310058, China

## Abstract

Fluorescent probes are one of the most popularly used bioimaging markers to monitor metabolic processes of living cells. However, long-term light excitation always leads to photobleaching of fluorescent probes, unavoidable autofluorescence as well as photodamage of cells. To overcome these limitations, we synthesized a type of photostable luminogen named TPE-TPP with an aggregation induced emission (AIE) characteristic, and achieved its three-photon imaging with femtosecond laser excitation of 1020 nm. By using TPE-TPP as fluorescent probes, three-photon microscopy under 1020 nm excitation showed little photo-damage, as well as low autofluorescence to HeLa cells. Due to the AIE effect, the TPE-TPP nanoaggregates uptaken by cells were resistant to photobleaching under three-photon excitation for an extended period of time. Furthermore, we demonstrated that for the present TPE-TPP AIE the three-photon microscopy (with 1020 nm excitation) had a better signal to noise ratio than the two-photon microscopy (with 810 nm excitation) in tissue imaging.

Fluorescent probes, with the advantages of high sensitivity, selectivity, and biocompatibility, have become powerful tools for bioimaging and biosensing^1^,^2^,^3^,^4^. Direct observation of events and metabolism by using fluorescent probes in individual cells or living organisms is very helpful to biomedical studies and applications^5^,^6^. In addition, fluorophores can also be conjugated with drugs or functional groups to achieve special functions^7^.

As a typical nonlinear optical effect, multiphoton absorption/luminescence has attracted much attention in various research areas, such as bioimaging and microfabrication^8^,^9^. Multiphoton luminescence occurs when the fluorophore is stimulated from the ground state to the excited state by absorbing two or more long-wavelength photons simultaneously, and then releases a short-wavelength photon^10^. Since long-wavelength (e.g. red and near infrared) photons have lower energy than short-wavelength (e.g. ultra-violet, blue and green) photons, multiphoton luminescence microscopy with red/near-infrared excitation produces less photo-damage to cells compared to (one-photon) confocal microscopy with ultra-violet/blue/green excitation. The linear absorption of endogenous fluorophores in cells is not negligible, but most of them have a relatively small cross section of two- or three-photon absorption, which gives the multiphoton microscopy the advantage of a low autofluorescence background and high imaging contrast. Furthermore, due to a nonlinear excitation mode of multiphoton microscopy, the fluorophores outside the beam focus cannot be excited, reducing the possibility of photobleaching of the fluorophores^11^.

Since multiphoton luminescence is a high-order nonlinear optical phenomenon, with the limitation of the excitation efficiency, fluorescent dyes with high concentration are usually required. However, high concentration of dye is always prone to aggregation, and charge transport is favored by dense ordered molecular stacking derived from strong π-π interactions^12^, which often leads to photoluminescence (PL) quenching. This is a concentration quenching phenomenon usually called Aggregation Caused Quenching (ACQ). Fortunately, luminogens with aggregation-induced emission (AIE) characteristics exhibit opposite phenomena: they are almost nonfluorescent when molecularly dissolved in an organic solution, but become highly emissive in the aggregate state, with fluorescence increasing along with the increase in fluorophore concentration^13^. Restriction of intramolecular motions (RIM) is proposed to be the main cause of the AIE effect^14^. Based on this mechanism, hydrophobic AIE dyes can be coated with various types of nanoparticles^8^,^15^, or form nanoaggregates spontaneously in an aqueous solution. The nanoprobes are very resistant to photobleaching even under high-power laser irradiation^16^.

Recently, we have reported a photostable AIE dye named tetraphenylethene-triphenylphosphonium (TPE-TPP). It carries two positive charges in each molecule, and forms nano-aggregates in cells, due to the negative membrane potential of cells^16^. TPE-TPP has short-wavelength fluorescence emission (with an emission peak at 466 nm), and the linear absorption in the near-infrared spectral region (e.g. 810 nm and 1020 nm) is ignorable, which makes it useful for multi-photon bioimaging with near infrared excitation.

In this manuscript, we realized multiphoton fluorescence imaging of living cells with TPE-TPP staining and studied the photostability of TPE-TPP under two-photon and three-photon excitation. Compared with two-photon imaging, three-photon imaging (with an excitation wavelength of 1020 nm) possesses the advantages of less photodamage to cells, lower autofluorescence, and higher spatial resolution. In addition, we also illustrated that TPE-TPP assisted three-photon microscopy possessed better signal to noise ratio than two-photon microscopy in tissue imaging.

## Results

### Multiphoton fluorescence of TPE-TPP

**Figure 1:**
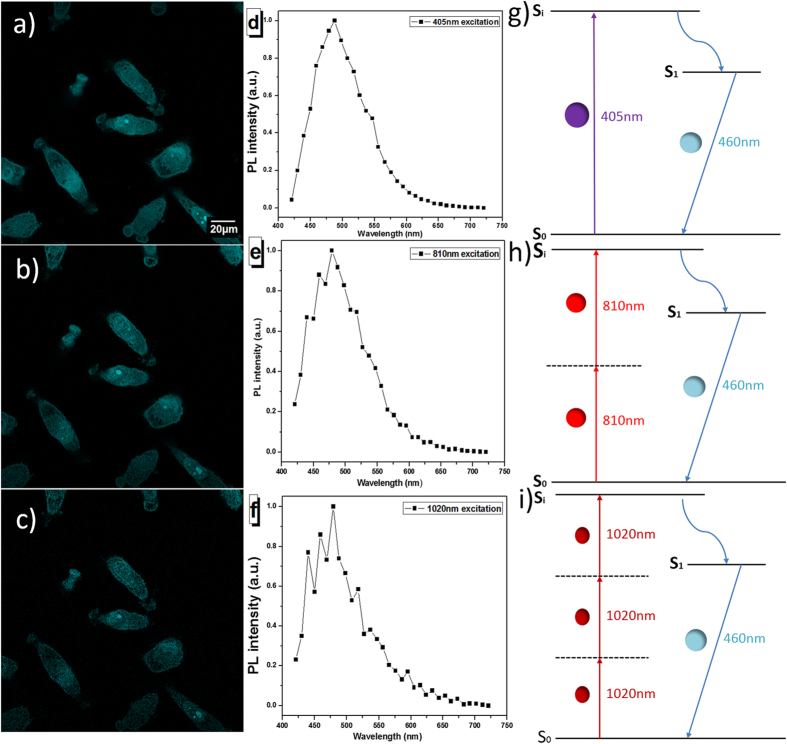
(**a–c**) One-, two- and three-photon microscopic images of TPE-TPP treated HeLa cells, with an excitation of (**a**) 405 nm-CW, (**b**) 810 nm-fs, and (**c**) 1020 nm-fs lasers. (**d**) one-, (**e**) two- and (**f**) three-photon fluorescence spectra of TPE-TPP labeled cells. (**g–i**) Diagrams showing the proposed mechanism for one-, two- and three-photon excitation and luminescence. S_i_ is the higher (electronic or vibronic) state; S_1_ is the lower radiative state; S_0_ is the ground state. Under our experimental conditions, luminescence occurred as a result of radiative transitioning between S_1_ and S_0_.

The chemical structure and absorption spectrum of TPE-TPP are shown (Figure S1 and S2, Supporting Information). Two triphenylphosphonium (TPP) groups are conjugated with a typical AIE luminogen called tetraphenylethene (TPE), granting the new molecule with AIE characteristics. The peak absorption wavelength of TPE-TPP is 320 nm, which is in the ultraviolet spectral range. The solid state of TPE-TPP showed a high absolute fluorescence quantum yield of 50.4%. We firstly performed one-photon confocal microscopic imaging of HeLa cells, which were treated with TPE-TPP. The final working concentration of TPE-TPP in cell dishes was 50 μM, and the CW excitation wavelength was 405 nm. As shown in Fig. 1a, the cells were uniformly covered by bright fluorescence, and its spectrum was shown in Fig. 1d, illustrating that TPE-TPP has effectively stained the cells. Since ultraviolet excitation is prone to doing harm to cells^17^, we consider using long-wavelength femtosecond (fs) laser excitation to carry out multiphoton fluorescence imaging of cells. TPE-TPP has negligible linear absorption at 810 nm and 1020 nm (Figure S2, Supporting Information), and these two wavelengths were selected for the two- and three-photon excitations of TPE-TPP molecules, respectively.

Figure 1b,c show the two- and three-photon images of TPE-TPP treated HeLa cells, from which we know that both the two- and three-photon fluorescence signals of TPE-TPP in the cells were very distinct. Figure 1d,e,f show the one-, two-, and three-photon fluorescence spectra from HeLa cells, and all of them have a similar luminescence spectrum, ranging from 425 to 600 nm and with a maximum at 470 nm. The fluorescence spectra from the cells are also similar to the three-photon fluorescence spectrum of TPE-TPP in aggregate state (solid), which was excited by a 1020 nm fs laser (Figure S3). This is because all the emissions originate from the same excited state to the same ground state^18^. As illustrated in Fig. 1h, the energy of one 810 nm-photon is not large enough to overcome the bandgap between the ground state (S_0_) and excited state (S_1_) of TPE-TPP, and thus emission of TPE-TPP under 810 nm-fs-excitation is induced by simultaneous absorption of two photons. Similarly, as shown in Fig. 1i, the energy of two 1020 nm-photons is also not large enough to overcome the bandgap between the ground state (S_0_) and excited state (S_1_) of TPE-TPP, so emission of TPE-TPP under 1020 nm-fs-excitation is induced by the simultaneous absorption of three photons^19^. We then measured the three-photon absorption coefficient of TPE-TPP molecules (10 mM in DMSO) at the wavelength of 1020 nm, which is about 4.08 × 10^−6^ cm^3^/GW^2^, and the corresponding three-photon cross-section was calculated to be 2.54 × 10^−80^ cm^6^ s^2^.

We further used TPE-TPP to treat two other types of cells. One is a kind of cancer cell called A549, and the other one is a kind of normal cell called BEAS-2b. TPE-TPP was successfully uptaken by these two kinds of cells, and the one-, two-, and three-photon fluorescence images of cells were shown in Figure S4.

### Photostability of multiphoton fluorescence

Resistance to photobleaching is a very important requirement for fluorescent probes. To test the photostability of TPE-TPP under various excitation modes, we chose a widely used commercial luminogen (Mito Tracker red FM, MT), which can also stain cells, as the control^20^,^21^. MT is a typical ACQ fluorophore that can emit bright two-photon fluorescence under 810 nm or 1020 nm fs excitation. We explored the photostability property of both TPE-TPP and MT under two-photon (with an 810 nm-fs laser) and three-photon excitation (with a 1020 nm-fs laser).

**Figure 2:**
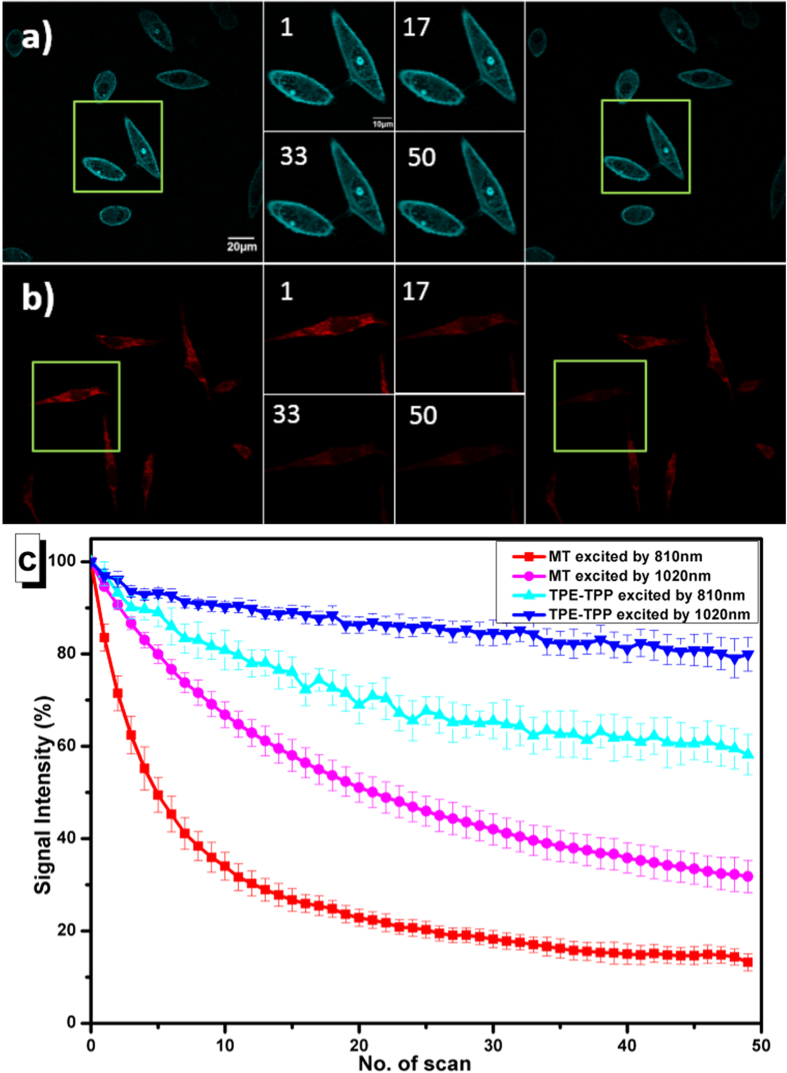
(**a,b**) Multiphoton fluorescence images of living HeLa cells targeted with (**a**) TPE-TPP and (**b**) MT, and excited by a 1020 nm fs laser. One area is chosen to be exposed to an increasing number of scans (the number of scans is shown in the upper left corner), and the whole view after photobleaching is also shown. (**c**) Signal loss (%) of fluorescent intensity of TPE-TPP and MT with increasing number of scans. Excitation wavelengths of fs laser: 810 nm (red curve for MT and cyan curve for TPE-TPP) and 1020 nm (purple curve for MT and blue curve for TPE-TPP); emission filter: 450–520 nm (for TPE-TPP) and 580–688 nm (for MT); irradiation time: 5.36 s/scan.

We first study the photostability of TPE-TPP and MT under 810 nm-fs-excitation . According to the absorption and emission spectra of TPE-TPP and MT (Figure S5, Supporting Information)^20^, we know there was a two-photon excitation effect for both types of dyes. Two dishes of HeLa cells were treated with 50 μM TPE-TPP and 50 nM MT for 2 h, respectively. A scanning confocal microscope (FV1000, Olympus) equipped with a wavelength-tunable fs laser was adopted to perform the multiphoton cell imaging. We can see very little fluorescence intensity change from TPE-TPP treated cells after 50 scans (5.36 s/scan, total irradiation time was ∼5 min, the power of 810 nm fs laser after a 60× objective lens was ~10 mW, Figure S6). However, fluorescence intensity change from MT treated cells was very obvious after 50 scans. The initial two-photon fluorescence intensity for the first scan of TPE-TPP and MT stained cells was used for normalization, and the percentage of signal loss after each additional scan was calculated. As shown in Fig. 2c, even during 50 scans, the signal loss of TPE-TPP was less than 40% (cyan curve). In contrast, the signal loss of MT reached 80% after 25 scans (red curve). We then study the photostability of TPE-TPP and MT under 1020 nm-fs-excitation with a laser power of ~20 mW after the 60× objective lens. In this case, there was a three-photon excitation effect for TPE-TPP while it was still a two-photon excitation effect for MT. As shown in Fig. 2a, the fluorescence loss from TPE-TPP stained cells could be hardly recognized after 50 scans. According to the blue curve in Fig. 2c, the signal loss of TPE-TPP under three-photon excitation was less than 20% even during 50 scans. However, the fluorescence loss from MT stained cells was still very distinct (Fig. 2b), and the signal loss of MT reached 55% after 25 scans (purple curve in Fig. 2c).

From the experimental results, we know TPE-TPP has better photostability than MT under both 810 nm and 1020 nm fs excitation. The AIE feature of TPE-TPP could explain this phenomenon. TPE-TPP was in the form of nanoaggregates when it was added into the cell dishes and then taken by the cells. The fluorescence from TPE-TPP nanoaggregates was much brighter than that from a single TPE-TPP molecule. During multiphoton excitation, the outermost layer of the nanoaggregates might be photobleached by the excitation light, while the nanoaggregates could prevent further photobleaching and photo-oxidation by preventing oxygen diffusion into the nanoaggregates^16^. Furthermore, it is worth noting that the photostability of TPE-TPP under three-photon excitation was higher than that under two-photon excitation. As three-photon absorption is a higher order nonlinear optical process than two-photon absorption, the actual experiment we set the excitation power of the 1020 nm fs laser (~20 mW) a bit higher than that of the 810 nm fs laser (~10 mW) to guarantee the same imaging effect under both two-photon and three-photon excitations. However, the signal loss of TPE-TPP under three-photon excitation (less than 20%) was still less than that of TPE-TPP under two-photon excitation (more than 35%) after 50 scans. The phenomenon can be attributed to the fact that 1020 nm photons have less energy than 810 nm photons, and the photo damage and photo-oxidation to TPE-TPP nanoaggregates under 1020 nm-excitation was less.

### Photodamage assessment of cells under 810 nm and 1020 nm fs excitation

**Figure 3:**
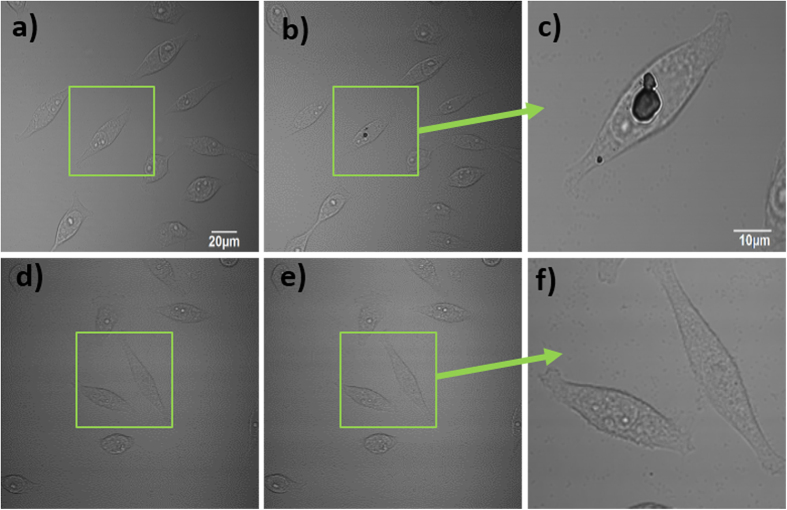
Transmission imaging of HeLa cells (**a,d**) before and (**b,e**) after one area is scanned by the 810 nm (**b**) and 1020 nm (**e**) fs laser for ~5 min. (**c,f**) Magnified areas scanned by the 810 nm (**c**) and 1020 nm (**f**) fs laser.

In spite of a higher excitation power of a 1020 nm fs (as compared with the excitation power of an 810 nm fs laser) during cell imaging, we found almost no photo-damage of cells under 1020 nm excitation. As shown in Fig. 3, after excited by the 810 nm-fs laser (~15 mW after the 60× objective lens, the imaging area was enlarged for 2 times via the software) for ~5 min, a burned spot was observed in the HeLa cell, illustrating that the laser beam did produce some photodamage to the cell. However, after being excited by the 1020 nm-fs laser (~30 mW after the 60× objective lens, the imaging area was enlarged for 2 times via the software) for the same period of time, no burned spot occurred, and the cells still looked very healthy. The burned spot may have resulted from multiphoton absorption of the cell and the intracellular destructive plasma formation under 810 nm excitation^22^. The energy of 1020 nm-photons is much less than that of 810 nm-photons, and consequently we could not observe the photodamage towards cells under 1020 nm-excitation.

### Spatial resolution of multiphoton microscopy under 1020 nm fs excitation

TPE-TPP-assisted three-photon fluorescence microscopy also possesses the advantage of high spatial resolution. Theoretically, for the same excitation wavelength, three-photon imaging is expected to achieve a higher spatial resolution of (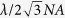), compared with one-photon imaging (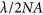) and two-photon imaging (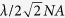)^23^. When we used a 1020 nm fs laser to perform multiphoton imaging, TPE-TPP underwent three-photon excitation while MT underwent only two-photon excitation. Thus, for MT, the theoretical spatial resolution under 1020 nm excitation is 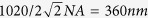 (NA=1.0). For TPE-TPP, the theoretical spatial resolution under 1020 nm excitation is 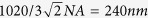. We further proved this theory in our multiphoton cell imaging experiment (Figure S7, Supporting Information).

### Signal to noise ratio of cell imaging under one-, two-, and three-photon excitation

Autofluorescence is always an unavoidable issue that exists in bioimaging. Under ultraviolet excitation, the intrinsic endogenous fluorophores in cells produce autofluorescence, whose typical spectra are mainly in the cyan, blue and green wavelength region^24^. TPE-TPP emits cyan light with an emission peak at 470 nm, and its spectrum is just located in the spectral region of intrinsic autofluorescence of the cell. In addition, both TPE-TPP and endogenous fluorophores have high absorption efficiency under ultraviolet excitation, which makes it difficult to distinguish the emission of TPE-TPP from the background autofluorescence during one-photon cell imaging. Fortunately, multiphoton excited imaging provides an approach to solve this problem.

**Figure 4:**
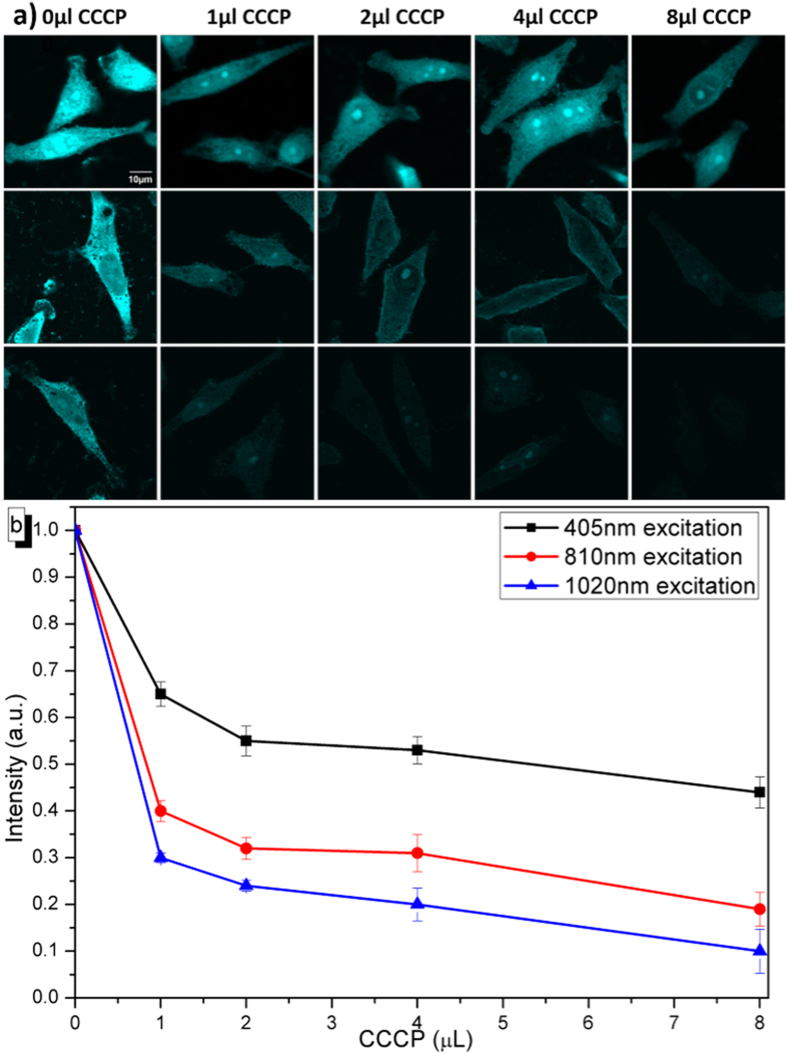
(**a**) Fluorescence imaging of TPE-TPP labeled HeLa cells, (cells have been treated with different dosages of CCCP, as shown on the top of the figures) excited by 405 nm CW (top row), 810 nm fs (middle row), and 1020 nm fs (bottom row) lasers, respectively. (**b**) Normalized fluorescence intensity of TPE-TPP stained cells with treatment of increasing dosages of CCCP, under one- (black curve), two- (red curve) and three-photon (blue curve) excitation.

The cell labeling capability of TPE-TPP nanoaggregates mainly results from their lipophilicity and electrophoretic force. Carbonyl cyanide m-chlorophenylhydrazone (CCCP) is a commonly used chemical protonophore that directly interferes with mitochondrial function and induces apoptosis by changing the mitochondria membrane potential (ΔΨm) of cells^25^. Thus, we can tune the uptake efficiency of TPE-TPP in cell by tuning the ΔΨm, which can be controlled by adding different quantities of CCCP in the cells. In our experiment, we treated cells with different dosages (0 μL, 1 μL, 2 μL, 4 μL and 8 μL) of CCCP (20 mM, in DMSO) on cell plates with a 2 mL culture medium for 30 min, and the cells were then incubated with TPE-TPP for 1 h. These samples were imaged with the aforementioned scanning confocal microscope, under the excitation of the 405 nm CW, 810 nm fs and 1020 nm fs laser, respectively. With the increasing CCCP, the TPE-TPP nanoaggregates were resistant to enter the cell, and a decreasing concentration gradient formed. As shown in Fig. 4a, the one-, two- and three-photon fluorescence intensity from TPE-TPP stained cells became gradually weaker when increasing the dosage of CCCP. The initial fluorescence intensity (under one-, two- and three-photon excitation) from TPE-TPP stained cells without CCCP treatment was used for normalization, and the fluorescence intensity of cells after the treatment of various CCCP dosages was calculated accordingly. Figure 4b shows that the fluorescence intensity of cells under one-photon (405 nm) excitation decreased only about 50% with inhibiting effect of 8 μL CCCP. Under two-photon (810 nm) and three-photon (1020 nm) excitation, fluorescence intensities decreased 75% and 90%, respectively, when 8 μL CCCP was added into the cells.

The different decrease tendencies of the fluorescence intensity under one-, two- and three-photon excitation could be attributed to the intrinsic background autofluorescence. As introduced above, the (one-photon) absorption of endogenous fluorophores in cells is not negligible. Under the 405 nm-CW-excitation, the fluorescence from TPE-TPP and the endogenous fluorophores were excited simultaneously. That’s why fluorescence could still be observed from the cells when the dosage of CCCP reached 8 μL, since most of them were from the autofluorescence. However, most endogenous fluorophores in the cells have relatively small two- and three-photon absorption cross sections, and it is somewhat difficult to excite the autofluorescence of the cells under 810 nm and 1020 nm fs excitation. For TPE-TPP, its two- and three-photon absorption efficiency is higher. Almost no multiphoton fluorescence could be observed from TPE-TPP stained cells when the dosage of CCCP was 8 μL, since the autofluorescence under multiphoton excitation was extremely weak. Multiphoton microscopy provides higher signal to noise ratio (SNR) of imaging, which can help us monitor tiny changes of TPE-TPP in cells without the influence of autofluorescence.

### *Ex vivo* three-photon fluorescence imaging of brain tissue stained with TPE-TPP

**Figure 5:**
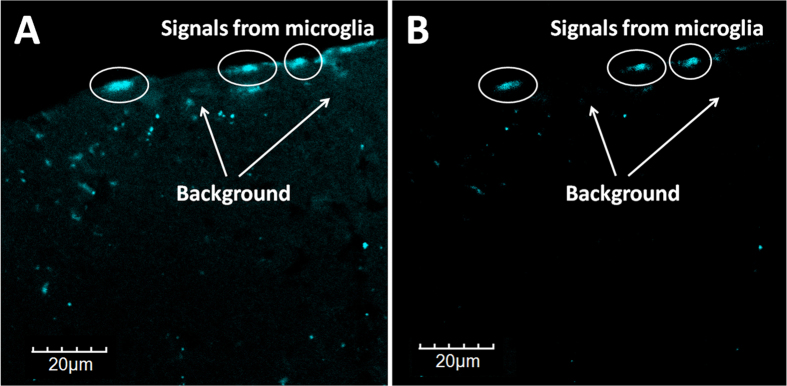
(**A**) Two-photon fluorescence imaging of brain tissue stained with TPE-TPP. Excitation: 810 nm fs laser. (**B**) Three-photon fluorescence imaging of brain tissue stained with TPE-TPP. Excitation: 1020 nm fs laser.

After using the TPE-TPP assisted three-photon microscopy for cell imaging, we further performed three-photon fluorescence imaging of tissue. Previously, we have demonstrated that TPE-TPP can be used to specifically target brain-microglia of mice through microinjection^26^. Figure 5B shows the typical three-photon fluorescence imaging (under a 1020 nm fs excitation) of a sliced brain tissue of a mouse, which was treated with TPE-TPP through microinjection. We could discriminate the staining of TPE-TPP in the microglia of the brain tissue very clearly with almost no background autofluorescence, illustrating the “signal to noise ratio” of the imaging was very high. It is worth noting that the average power of the adopted 1020 nm fs excitation was not high, and 1020 nm-photon energy was lower than 810 nm-photon energy, thus no thermal damage towards tissues was observed during three-photon *ex vivo* imaging process. For comparison, both the two-photon fluorescence of TPE-TPP and two-photon autofluorescence from the brain tissue were visualized, under the 810 nm-fs laser excitation. The autofluorescence spots, indicated by the arrows in Fig. 5A, made the identification of the TPE-TPP molecules (through two-photon fluorescence) very difficult. Hence, we conclude that longer wavelength excited three-photon microscopy is more suitable for high-contrast tissue imaging.

## Discussion

We have synthesized a special AIE luminogen that is capable of labeling cell and emitting distinct two-photon and three-photon fluorescence under fs laser excitation. We have demonstrated that three-photon microscopy under 1020 nm excitation has the advantages of better photostability upon long-term light irradiation, less photo-damage, better spatial resolution, and higher SNR (free of autofluorescence), by utilizing TPE-TPP as fluorescent probes. Furthermore, we have achieved *ex vivo* three-photon fluorescence microscopic imaging of brain tissues of mice, which were stained with TPE-TPP, illustrating that for this TPE-TPP AIE the three-photon microscopy possesses better SNR than the two-photon microscopy in tissue imaging. TPE-TPP based three-photon microscopy could be a promising tool for biomedical detection and diagnosis.

## Methods

### Materials

Dimethylsulfoxide (DMSO) was obtained from Sinopharm Chemical Reagent Co., Ltd. Mito Tracker Red FM was purchased from Invitrogen. Carbonyl cyanide m-chlorophenylhydrazone (CCCP) was purchased from Sigma. Cell-culture products, unless otherwise mentioned, were purchased from Invitrogen Gibco. TPE-TPP was synthesized following our previous work^16^.

### Quantum yield measurement

The absolute quantum yield of solid TPE-TPP was measured with a commercial system (C11347-11Quantaurus-QY, HAMAMARSU).

### Cell culture

HeLa cells were cultivated in Dulbecco minimum essential media (DMEM) with 10% fetal bovine serum (FBS), 1% penicillin, and 1% amphotericin B, at 37 °C and 5% CO_2_.

### Cell imaging

HeLa cells were grown overnight on 35 mm petri dishes with cover slips. The live cells were treated with 50 μM of TPE-TPP for 30 min (by adding 2 μL of a 50 mM stock solution of TPE-TPP in DMSO to 2 mL culture medium) or 50 nM MitoTracker® Red FM (MT) for 30 min (by adding 0.5 μL of a 200 μM stock solution of MT in DMSO to 2 mL culture medium). The cells were observed by an upright Olympus laser scanning confocal microscope (FV1000) equipped with CW lasers with various wavelengths and a wavelength-tunable fs laser, which can achieve one-photon (405 nm CW), two-photon (810 nm fs) and three-photon (1020 nm fs) fluorescence imaging.

### Imaging of brain tissue of mice

All the animal experiments were performed in compliance with the Zhejiang University Animal Study Committee’s requirements for the care and use of laboratory animals in research. The animal housing area (located in Animal Experimentation Center of Zhejiang University) was maintained at 24 °C with a 12 hour-light/dark cycle, and animals were fed with water and standard laboratory chow. 8-week-aged female BALB/c mice were used for brain imaging. After anesthetized with pentobarbital, the skulls of the mice were opened up through microsurgery. A 0.2 μL stock solution of TPE-TPP in DMSO was microinjected into the brain of mice. The mice were then sacrificed, and their brain tissues were taken out. The brain tissues were fixed, sectioned, and further imaged with the upright confocal microscope for two/three-photon fluorescence observation. The wavelength of the excitation fs laser was 810 nm and 1020 nm, respectively.

All the protocol of animal experiments was approved by the Institutional Ethical Committee of Animal Experimentation of Zhejiang University in China, and the experiments were performed strictly according to governmental and international guidelines on animal experimentation. According to requirements for Biosafety and Animal Ethics, all efforts were made to minimize the number of animals used and their suffering.

## Additional Information

**How to cite this article**: Zhu, Z. *et al.* Using AIE Luminogen for Long-term and Low-background Three-Photon Microscopic Functional Bioimaging. *Sci. Rep.* **5**, 15189; doi: 10.1038/srep15189 (2015).

## Supplementary Material

Supplementary Information
