# Supplementary material for: Using AIE Luminogen for Long-term and Low-background Three-Photon Microscopic Functional Bioimaging
Source: Sci Rep. 2015 Oct 15;5:15189. doi: 10.1038/srep15189 (PMC4606727; doi:10.1038/srep15189)
Supplement: Supplementary Information [file srep15189-s1.doc]

Supporting Information

**Using AIE luminogen for Long-term and Low-background Three-Photon Microscopic Functional Bioimaging**

*Zhenfeng Zhu,* ^†^ *Chris W. T.* *Leung,* ^‡^ *Xinyuan Zhao,*^§^ *Yalun Wang,*^†^ *Jun Qian,*,*^†^ *Ben Zhong Tang,*^‡^ *and Sailing He**^,†^

^†^State Key Laboratory of Modern Optical Instrumentation (Zhejiang University), Centre for Optical and Electromagnetic Research, Zhejiang Provincial Key Laboratory for Sensing Technologies, JORCEP (Sino-Swedish Joint Research Center of Photonics), Zhejiang University, Hangzhou, 310058, China

^‡^Department of Chemistry, The Hong Kong University of Science & Technology, Clear Water Bay, Kowloon, Hong Kong, China

^§^Bioelectromagnetics Laboratory, Zhejiang University School of Medicine, Hangzhou 310058, China


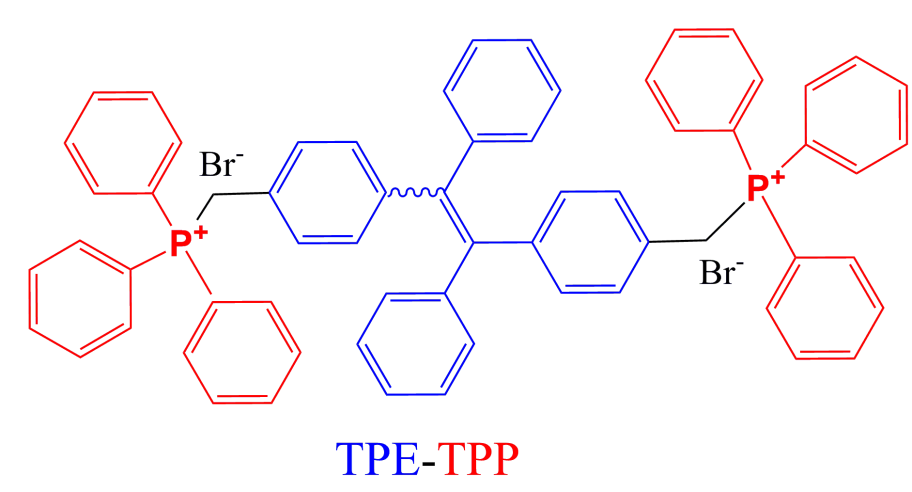


**Figure S1.** Chemical structure of TPE-TPP.


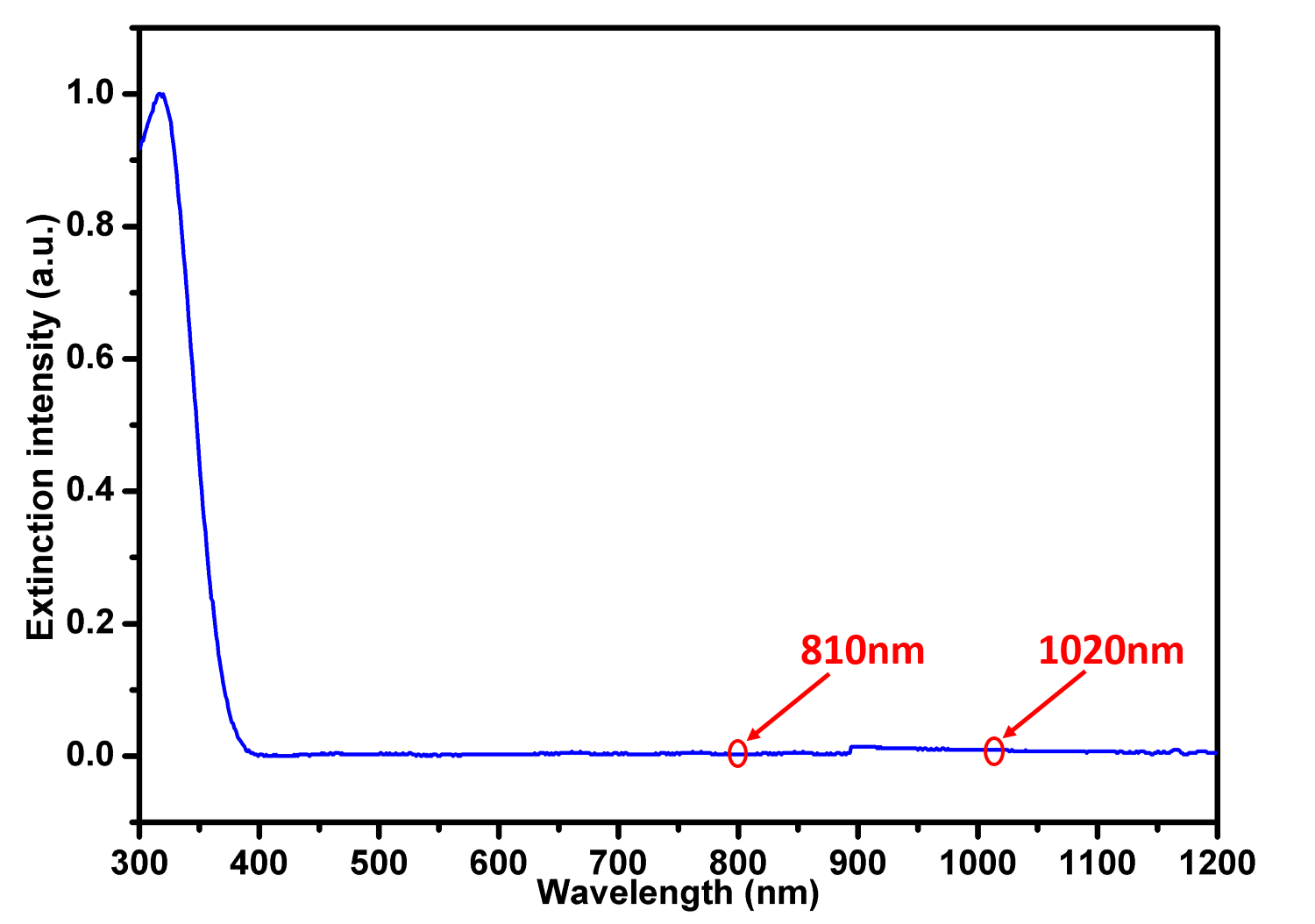


**Figure S2.** Absorption spectrum of TPE-TPP in DMSO

**Experimental set-up for the measurement of three-photon fluorescence of TPE-TPP in solid state.**

The experimental set-up has been reported in our previous works.^[3]^ Beam from the 1020 nm fs laser passed through a long-pass filter and a 1/4 waveplate, and was coupled into an objective (10×, NA=0.25) to achieve high excitation intensity towards the TPE-TPP sample on a glass slide. The excited signals were collected forward by another objective (10×, NA=0.25) and recorded with an optical fiber spectrometer (PG 2000, Ideaoptics Instruments), after passing through a short-pass filter.


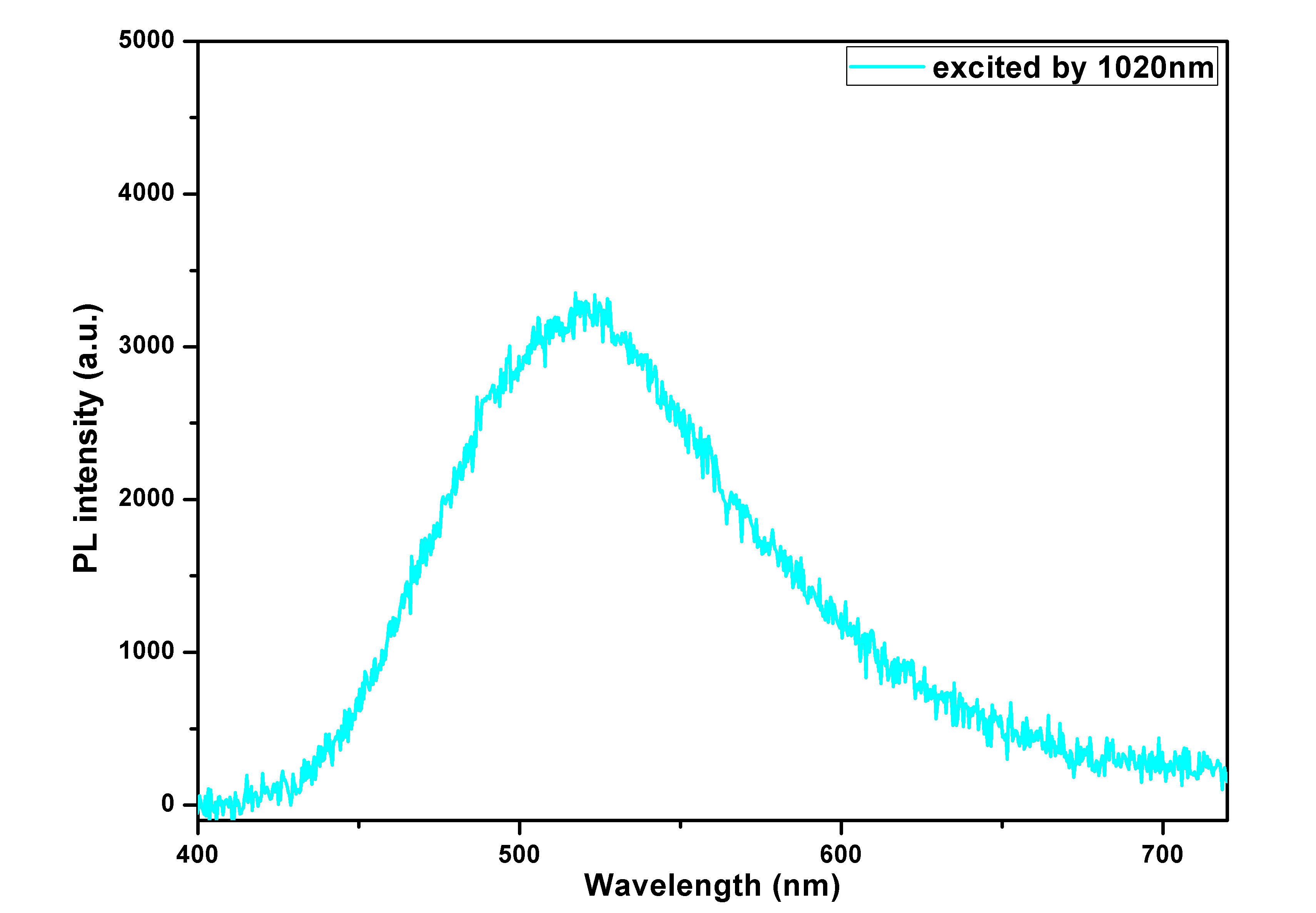


**Figure S3.** Three-photon fluorescence spectrum of TPE-TPP in aggregate state, which was excited by a 1020 nm fs laser.

**Measurement of three-photon absorption cross-section of TPE-TPP.**

We used the nonlinear transmissivity measurement to calculate the three-photon absorption cross-section of TPE-TPP molecule at the wavelength of 1020 nm. The nonlinear transmissivity of a three-photon absorbing medium can be described as^3^

$$T(I_{0},\lambda)=\frac{1}{\sqrt{1+2\gamma(\lambda)I_{0}^{2}L}}$$

where I_0_ is the peak intensity of the incident light, L is the path length of the sample, γ(λ) is the three-photon absorption coefficient (in units of cm^3^/GW^2^) which is a macroscopic parameter depending on the concentration of the three-photon absorbing molecules. It can be further expressed as

$$\gamma(\lambda)=\sigma_{3}^{'}(\lambda)N_{0}=\sigma_{3}^{'}(\lambda)N_{A}d_{0}\times10^{-3}$$

where N_0_ is the molecular density (in units of 1/cm^3^), N_A_ is Avogadro’s number, and d_0_ is the molar concentration of the absorbing molecules (in units of M). Here σ'_3_(λ) is the molecular three-photon absorption cross-section (in units of cm^6^/GW^2^). There is also a parallel definition of the three-photon absorption cross-section, i.e., σ'_3_(λ)= σ_3_(λ)·(hν)^2^ (σ_3_(λ) is in units of cm^6^s^2^). To measure the three-photon absorption coefficient γ(λ) of TPE-TPP at 1020 nm, for which its linear (one-photon) attenuation is very small, we performed nonlinear transmission measurements based on a fs OPA system (Libra–USP-HE, 1 KHz, 160 fs). The laser beam was focused onto a 1-cm long cuvette containing 10 mM TPE-TPP in DMSO, and the transmissivity of 1020 nm fs laser passing through the TPE-TPP sample was measured accordingly.


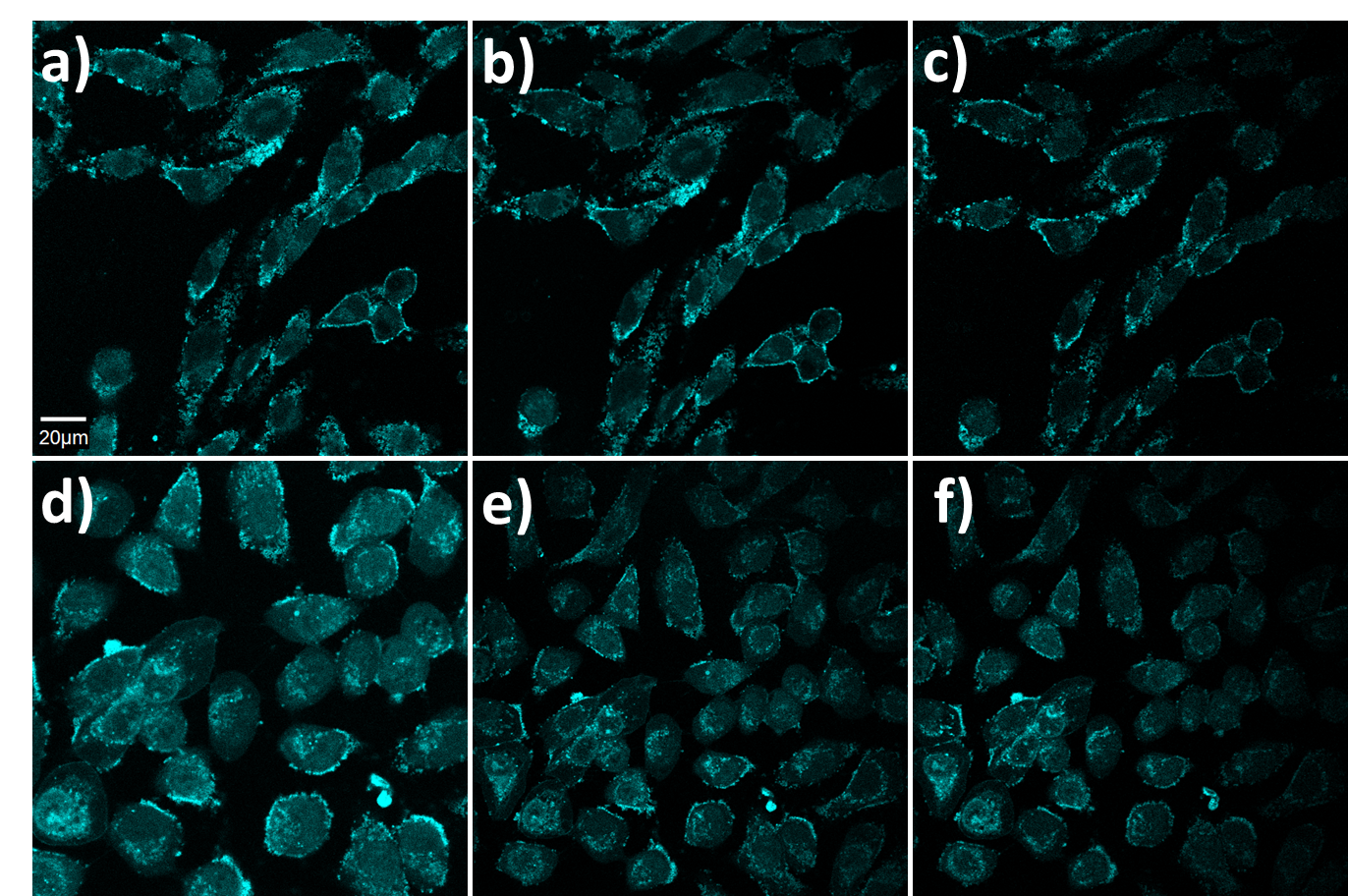


Figure S4. One-, two- and three-photon microscopic images of living (a-c) BEAS-2b and (d-f) A549 cells stained with TPE-TPP, which were excited by a 405 nm CW laser (a, d), a 810 nm fs laser (b, e), and a 1020 nm fs (c, f) laser, respectively.

**Mechanism of MT excited by 800 nm and 1020 nm fs laser.** According to the absorption and emission spectra of MT (Fig. S5), 810 nm is much longer than the emission wavelength of MT, so the energy of one 810 nm-photon is not large enough to overcome the bandgap between the ground state (S_0_) and excited state (S_1_) of MT. Thus, the emission of MT under 810 nm-fs-excitation is induced by simultaneous absorption of two 810 nm-photons. For 1020 nm-fs-excitation, half of its wavelength (510 nm) locates in the absorption spectrum region of MT, so the energy of two 1020 nm-photons is also large enough to overcome the bandgap between the ground state (S_0_) and excited state (S_1_), which means the emission of MT under 1020 nm-fs-excitation is a two-photon process as well.^2^


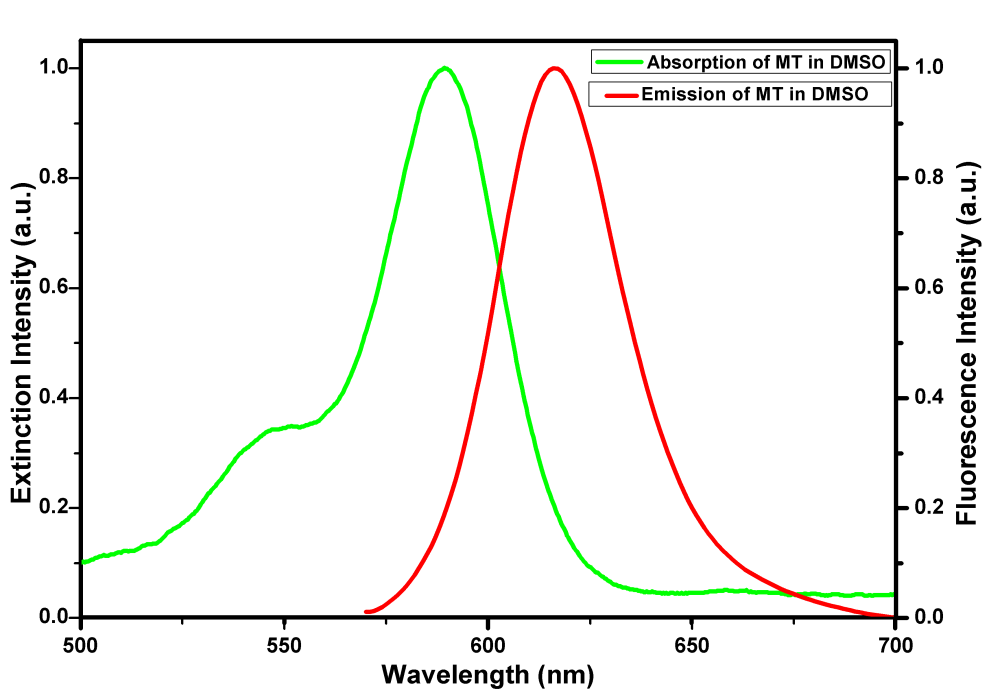


**Figure S5.** Absorption and fluorescence spectra of MT in DMSO


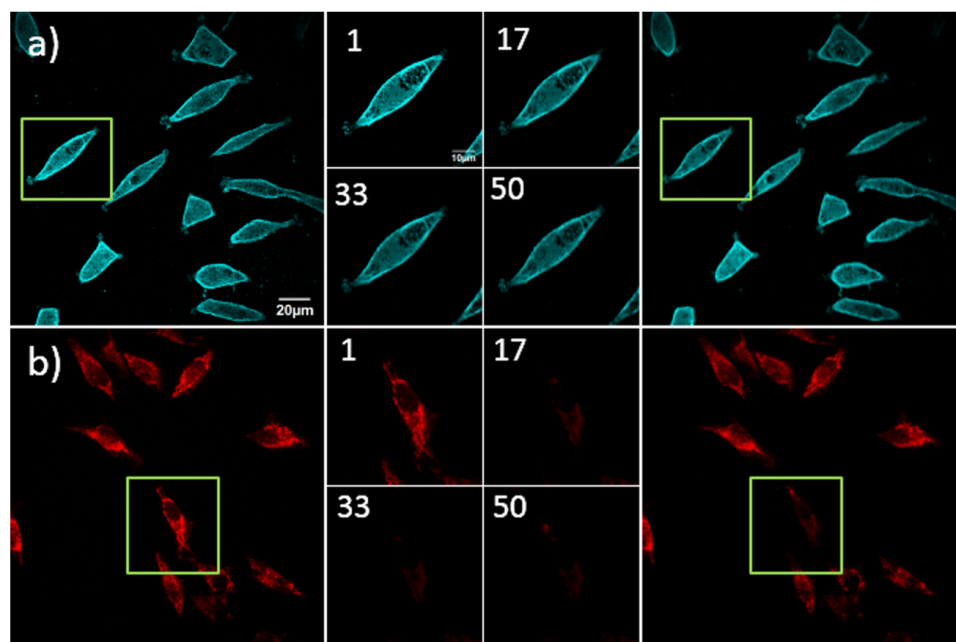


**Figure S6.** Multiphoton fluorescence images of living HeLa cells targeted with (a) TPE-TPP and (b) MT, excited by a 810 nm fs laser, one area is chosen to suffer with increasing number of scanning (the number of scans is shown in the upper left corner of each image), and the whole view after photobleaching is also shown. Excitation wavelength: 810 nm fs laser; emission filter: 450−520 nm (for TPE-TPP) and 580−688 nm (for MT); irradiation time: 5.36 s/scan.

**Spatial resolution of TPE-TPP and MT under the excitation of a 1020 nm fs laser.** Cells were stained with both MT and TPE-TPP. One area of the multiphoton images of cells are magnified by 20 times, as shown in Figure S7 (b-d). According to the multiphoton fluorescence signals from the same region of the cell in two different channels (cyan for TPE-TPP and red for MT), we know the spatial FWHM (full width at half maximum) in cyan channel is narrower than that in red channel (0.7 μm V. S. 1.15 μm), which illustrated TPE-TPP-assisted three-photon imaging has higher spatial resolution than MT assisted two-photon imaging.


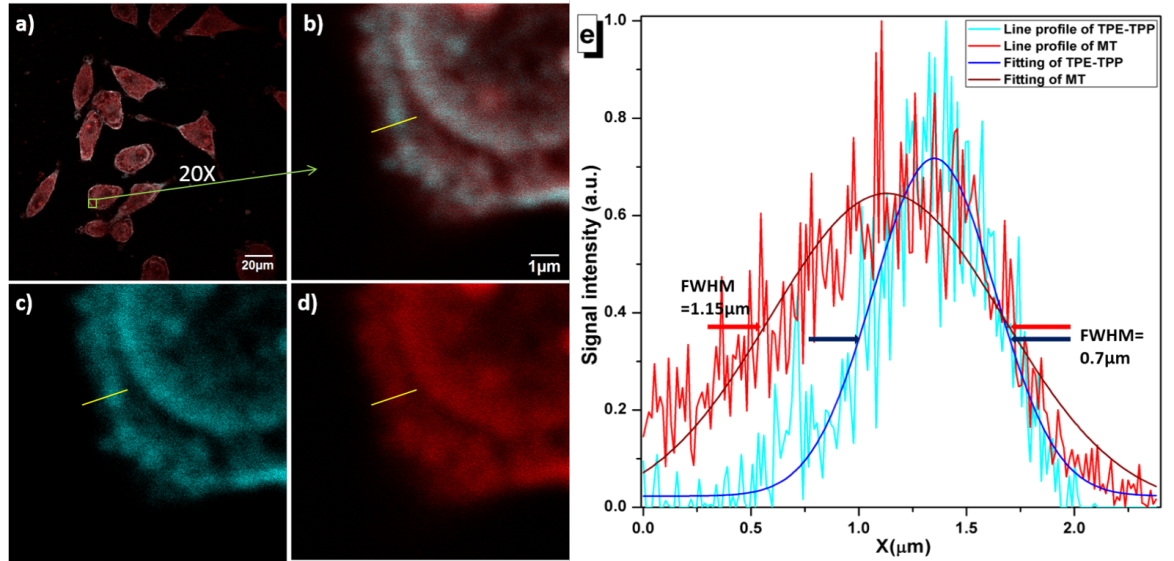


**Figure S7.** a) Multiphoton fluorescence imaging of Hela cells targeted by both TPE-TPP and MT, excited by a 1020 nm fs laser. Emission filter: 450−520 nm (cyan channel, for TPE-TPP), 580−688 nm (red channel, for MT). b-d) Magnified images (20×) of the outlined region in a. b) Merged channel, c) Cyan channel, d) Red channel. e) Line profiles of the spatial signal intensity from c (cyan) and d (red). Gaussian fits (purple for c and blue for d) are shown for clarity.

**References**

(1) Qian, J. et al. High-Order Non-linear Optical Effects in Organic Luminogens with Aggregation-Induced Emission. *Adv. Mater.* **27,** 2332-2339 (2015).

(2) Zheng, Q. D. et al. Frequency-upconverted Stimulated Emission by Simultaneous Five-photon Absorption. *Nat. Photonics.* **7,** 234-239 (2013).

(3) He, G. S. et al. “Multiphoton Absorbing Materials: Molecular Designs, Characterizations, and Applications”, *Chem. Rev.* **8,** 2688 (2008).
